# Twentieth Annual Meeting of the Mississippi Valley Association of Dental Surgeons

**Published:** 1864-04

**Authors:** 


### Proceedings of Societies.

### TWENTIETH ANNUAL MEETING OF THE MISSISSIPPI VALLEY ASSOCIATION OF DENTAL SURGEONS.

The Association met according to adjourment in the Lecture Room of the Ohio College of Dental Surgery, February 17th, 1864, 2 P. M.; the President, Dr. E. Collins in the Chair.

Members present; Drs. Geo. Watt, James Taylor, A. Berry, G. W. Keely, J. Taft, H. R. Smith, W. H. Shadoan, McCullum, J. P. Ullery, S. Driggs, Geo. F. Foote, James Leslie, A. S. Talbert, H. A. Smith, and Chas. James.

The minutes of the last meeting were read and approved. Two members of the Executive Committee being absent, Drs. Watt and James were on motion appointed in their stead. The Committee submitted the following report:

The Executive Committee would respectfully report the following as the order of business:

1. Report of Officers.
2. Election of Officers to take place at 10 o'clock A. M., on the 18th, to morrow.
3. Reading Essays.
4. Miscellaneous business. Reports of Committees in order at any interval in discussions or other business.

### SUBJECTS FOR DISCUSSION.

1. Anæsthetics.
2. Filling Teeth—material and mode of using.
3. The Office, its furniture and fixtures.
4. Pivot Teeth.

H. McCallum,

C. H. James,

Geo. Watt. } Committee.

The first subject for discussion was then taken up.

On motion adjourned to meet at 7 o'clock, P. M.

EVENING SESSION.

Minutes read and approved.

The Committee on membership presented the names of the following persons for membership:—Drs. J. Cheesebrough, A. Feeman, Wm. Taft, W. A. Morrison, J. H. McKellops, N. W. Williams, Geo. D. Belden, H. A. Beeman, all of whom were unanimously elected members of the Association.

The first subject was continued in discussion.

On motion adjourned to meet at 9 o'clock on Thursday.

### SECOND DAY.

MORNING SESSION.—Met at 9 o'clock, mintues read and approved.

Dr. Taft offered the following resolution:

1. *Resolved,* That a gold medal not exceeding $100 in value be awarded by the Association for the best Essay on Anæsthetics, to be approved by a committee of the Association.

2. That essays competing for prize must be placed in the hands of the committee as early as January 1st, 1865.

3d. That the committee have power to reject all essays presented, if regarded as unworthy of the award.

4. That rejected essays be promptly disposed of by the committee as directed by the authors.

5. That when the committee has made the award, the approved essay be sealed up, and thus preserved till the committee report to the Association, when it and the envelope containing the authors name are to be opened.

6. That the copy-right shall belong to the Association.

7. That the committee shall not permit any one to inspect or see the copy of any essay till after making a report to the Association.

8. That the committee report at the next annual meeting.

On motion the consideration of this resolution was laid over till the afternoon session.

The time appointed for the election of officers having come, the Association proceeded to the election with the following result:—

*President*—Dr. A. Berry.

*Vice-President*—Dr. G. W. Keely.

*Recording Secretary*—Dr. Geo. F. Foote.

*Corresponding Secretary*—Dr. H. A. Smith.

*Treasurer*—Dr. J. Taft.

*Executive Committee*—Drs. J. Cheesebrough, H. A. Smith, and A. S. Talbert.

The following named persons were chosen as Delegates to the American Dental Association:—Drs. Geo. D. Belden, W. H. Shadoan, J. Cheesebrough, McCallum, Geo. F. Foote, and S. Driggs.

On motion it was,

*Resolved*, That the President of the Association and Delegates present, shall have power to fill vacancies, if any occur in the delegation to the American Dental Association.

The second subject was then continued in discussion till the hour for adjournment. Adjourned to meet at 2 o'clock, P. M.

AFTERNOON SESSION.

Minutes read and approved. President elect, Dr. A. Berry, in the Chair.

The Treasurer, Dr. Taft, presented the following report:

Dr Taft in account with the Mississippi Valley Association of Dental Surgeons. Dr.

| | |
|---|---|
| To amount balance in Treasury at last report | $ 5 68 |
| To amount received, annual dues at meeting, 1863 | 15 00 |
| To Initiation Fees | 6 00 |
| | $26 68 |
| Cr by amount paid Janitor | 5 00 |
| Balance | 21 68 |

Drs. Watt and Cheesebrough were appointed auditing committee on the above and reported as follows:

Having examined the Treasurer's report, we find it correct.

G. WATT,<br>J. CHEESEBROUGH, } Committee.

An essay was read by Dr. Shadoan, subject filling teeth. Second subject continued in discussion.

Dr. Taft's resolution to award a prize for the best essay on anæsthetics was then taken up and passed, and Drs. J. Taft, G. Watt, Jas. Taylor, Geo. Foote, and A. Berry appointed the committee to make the award.

On motion adjourned to meet at 7½ o'clock, P. M.

EVENING SESSION.

Association met according to adjournment, Dr. Berry in the Chair. In absence of the Secretary, Dr. Cheesebrough was apponted Secretary *pro tem.*

Committee on miscroscope made a report, which on motion was approved and adopted.

On motion the former committee on microscope was continued.

It was moved and carried that a committee on publication be appointed to take the place of the committee on essay; Drs. Berry, Collins, and H. A. Smith were appointed that committee.

Dr. Taft proposed to the society that his intention was to furnish the society a silver medal to be given to the person that makes the most important invention, discovery, or improvement during the next year.

On motion, the proposition of Dr. Taft was accepted by the society.

On motion, it was adopted that the society appoint a committee of three to report to this society, at its next session, the greatest invention, discovery or improvement.

Drs. Talbert, Shadoan, and Driggs were appointed that comm'ttee.

Dr. Mullet, of Vevay, Indiana, through Mr. James Leslie, tendered to the society and profession a universal drill.

On motion, it was adopted that the society accept Dr. Mullet's proposition, and tender him a vote of thanks for his very ingenious instrument.

The discussion of the next subject in order being announced.

Moved and adopted to adjourn till to-morrow at 2 P. M.

AFTERNOON SESSION—FRIDAY.

Association met, the President in the Chair. The fourth subject for discussion was taken up.

Dr. Talbert, read an essay, subject, Professional Elevation, which was on motion referred to the committee on publication.

The following resolution was passed:

*Resolved*, That members indebted for annual dues, be requested to meet such liabilities promptly, in person or by mail, that the Association may be able to meet its engagements in reference to its proposed prize for essay.

The Treasurer was on motion instructed to pay the Janitor the sum of eight dollars ($8,00) for services during the meeting of the Association.

On motion, adjourned to meet in the Dental College, February 1865, at 10 o'clock, A. M.

